# Perovskite quantum dot one-dimensional topological laser

**DOI:** 10.1038/s41467-023-36963-6

**Published:** 2023-03-15

**Authors:** Jingyi Tian, Qi Ying Tan, Yutao Wang, Yihao Yang, Guanghui Yuan, Giorgio Adamo, Cesare Soci

**Affiliations:** 1 Centre for Disruptive Photonic Technologies, TPI, Nanyang Technological University, 21 Nanyang Link, Singapore, 637371 Singapore; 2 Division of Physics and Applied Physics, School of Physical and Mathematical Sciences, Nanyang Technological University, Singapore, 637371 Singapore; 3 Energy Research Institute @NTU (ERI@N), Interdisciplinary Graduate School, Nanyang Technological University, 50 Nanyang Drive, Singapore, 637553 Singapore; 4 Present Address: College of Information Science and Electronic Engineering, Zhejiang University, Hangzhou, 310027 China; 5 Present Address: Department of Optics and Optical Engineering, University of Science and Technology of China, Hefei, 230026 China

**Keywords:** Microresonators, Semiconductor lasers

## Abstract

Various topological laser concepts have recently enabled the demonstration of robust light-emitting devices that are immune to structural deformations and tolerant to fabrication imperfections. Current realizations of photonic cavities with topological boundaries are often limited by outcoupling issues or poor directionality and require complex design and fabrication that hinder operation at small wavelengths. Here we propose a topological cavity design based on interface states between two one-dimensional photonic crystals with distinct Zak phases. Using a few monolayers of solution-processed all-inorganic cesium lead halide perovskite quantum dots as the ultrathin gain medium, we demonstrate a lithography-free, vertical-emitting, low-threshold, and single-mode laser emitting in the green. We show that the topological laser, akin to vertical-cavity surface-emitting lasers (VCSELs), is robust against local perturbations of the multilayer structure. We argue that the design simplicity and reduction of the gain medium thickness enabled by the topological cavity make this architecture suitable for low-cost and efficient quantum dot vertical emitting lasers operating across the visible spectral region.

## Abstract (web summary)

Topological lasers often suffer from low directionality, and/or complex design requirements hindering operation at small wavelengths. Here, by using a few monolayers of perovskite quantum dots, the authors demonstrate a lithography-free, vertical-emitting, single-mode laser emitting in the green.

## Introduction

The concept of topological photonics^1–3^, characterized by global topological invariants of the photon wavefunction in the optical dispersion bands, has been used in recent years for the design of optical cavities that are robust against fabrication imperfections, leading to the demonstration of several topological micro/nanolasers operating in the infrared^4–12^. The cavity design of these lasers relies on the excitation of boundary states (e.g., edge or corner states) at the interface of photonic structures in different topological phases. The difference of topological invariants across the interface defines the boundary states while the so-called bulk-edge correspondence underpins their topological protection^1,13–16^. Typical edge/corner emitting topological lasers based on 1D chains^6^ or 2D arrays^9^ of semiconductors resonators bear poor directionality, are difficult to outcouple, and are limited to infrared operation by their design complexity. Recently, topological lasers based on topological bulk bands^12^ and topological vertical-cavity laser arrays^10^ addressed the directionality and outcoupling issues but did not improve on design complexity and access to short emission wavelengths.

Here we demonstrate a lithography-free topological laser with interface states confined between two 1D photonic crystals with distinct Zak phases (i.e., the geometric phase picked up by a photon moving across the one-dimensional Brillouin zone)^13,17^. Solution-processed films of green-emitting halide perovskite quantum dots are used as gain media due to their large optical absorption cross-section, large exciton binding energy, high photoluminescence quantum yield, high defect tolerance, and compositional tunability^18–21^. Thanks to the topological cavity design, the microlaser with ultrathin gain medium (<50 nm) yields single-mode emission with a low lasing threshold (6.8 µJ/cm^2^) and is robust against local perturbations of the multilayer structure. By varying the gain medium thickness, the laser emission can be tuned continuously across the gain spectrum (from *λ* = 532 to 519 nm). This is particularly suitable for the realization of quantum dots lasers, as it allows overcoming issues of poor uniformity, clustering and luminescence quenching typical of thick quantum dot films. Moreover, this microlaser design might be beneficial for the realization of electrically pumped VCSELs, where the electrical conductivity of thick gain media is typically limited by carrier diffusion length.

## Results

### Design principles

**Fig. 1:**
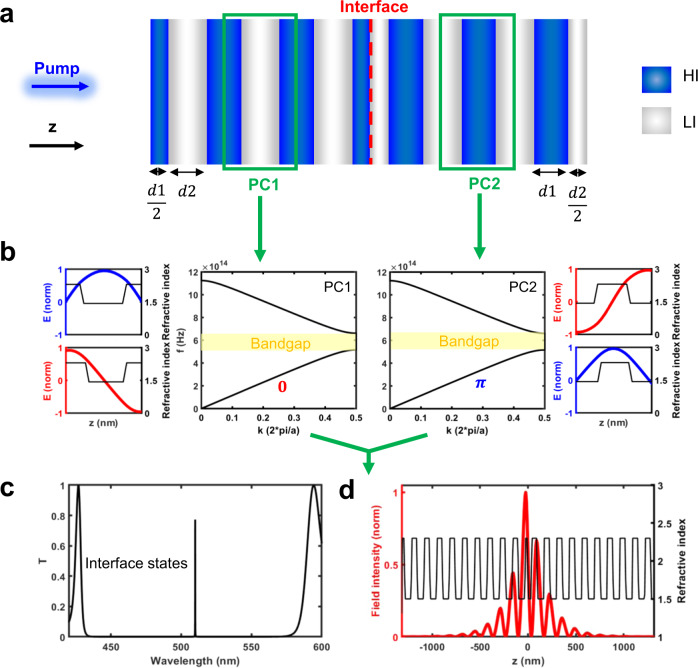
Design principles of a 1D topological microcavity. **a** Schematic of a one-dimensional (1D) topological microcavity formed by interfacing two photonic crystals (PC1 and PC2) composed of alternative high index (HI) and low index (LI) layers. The inversion center of PC1 is chosen to be within the LI layer, while the inversion center of PC2 is within the HI layer, as highlighted by the green boxes. **b** The calculated optical band structures of PC1 and PC2 are identical but have distinct Zak phases. The normalized electric field distributions along the z-axis within the respective unit cells for the first two optical bands are shown in the insets. **c** Calculated transmission spectra of the 1D topological cavity formed by 10 unit cells on each side of the interface, showing the appearance of a high-Q interface state within the optical bandgap. **d** Calculated spatial distribution of the electric field within the microcavity.

The existence of localized optical states at the interface between two 1D binary photonic crystals (PCs)^22,23^ is determined by their surface impedance mismatch. This is related to the geometric phases (i.e., the Zak phases) of the bulk optical bands lying below the PC bandgap on either side of the interface. The excitation of optical states confined near the interface is possible when the two PCs have surface impedances of opposite sign. In such systems with inversion symmetry, the Zak phase is a quantized topological invariant with value of 0 or $\pi$, corresponding to opposite parity of the optical modes with respect to the inversion center. For instance, the planar topological cavity depicted in Fig. 1a comprises of the interface between two semi-infinite 1D binary photonic crystals (PC1 and PC2) with inversion symmetry along the *z-*direction. The PCs consist of alternating low index (LI, with thickness of *d*_*1*_) and high index (HI, with thickness of *d*_*2*_) layers, at the middle of which lay the inversion centers of PC1 and PC2, respectively.

To minimize the cavity thickness, the topological cavity in our demonstration is designed to work within the first optical bandgap of a TiO_2_/SiO_2_ multilayer, with *n*_*HI*_ = 2.3, *n*_*LI*_ = 1.5, *d*_*1*_ = 62 nm, *d*_*2*_ = 70 nm. Although PC1 and PC2 share the same bulk optical bands, they carry distinct Zak phases for the lowest band due to the different placement of LI and HI about their inversion centers (Fig. 1b). Analytically, this is seen in the definition of the Zak phase ${\theta }_{{Zak}}$ carried by the lowest band^13^:

1 $${{\exp }}\left(i{\theta }_{{Zak}}\right)={{{{{\rm{sign}}}}}}\left[1-\frac{{\mu }_{s}{\varepsilon }_{c}}{{\mu }_{c}{\varepsilon }_{s}}\right]={{{{{\rm{sign}}}}}}\left(\eta \right)$$

Where $\eta$ is the surface impedance, *ε*_c_ and ${\mu }_{c}$ indicate the relative permittivity and permeability of the layer containing the inversion center and ${\varepsilon }_{s}$ and ${\mu }_{s}$ those of the side layers. From Eq. 1, PC1 and PC2 have Zak phase of 0 and $\pi$ and odd and even electric field distribution across their inversion centers (insets of Fig. 1b), respectively. Thus, surface impedances of PC1 and PC2 have opposite sign and an interface state exists within the first bandgap of the proposed 1D topological cavity.

The 1D topological cavity, consisting of two half-cavities of 10 unit cells, is expected to show a high-quality interface state (Q > 5000) around 515 nm, at the center of the first optical bandgap, as predicted by the calculated transmission spectrum shown in Fig. 1c. The electric field is highly confined at the interface of the two PCs, with an asymmetric distribution peaking inside the first HI layer of PC1 (Fig. 1d).

In view of employing this cavity design in a laser, we analyzed the robustness of the topological interface state against local perturbations of the lattice^6^ by substituting the first HI layer (*d* = 31 nm) near the interface with an hypothetical gain medium of refractive index *n*_*g*_ = 2.3 (losses are neglected in first approximation) and thickness varying by $-10{nm}\le \delta \le 10{nm}$ $(-40\%\le \delta /d\le 40\%)$.

**Fig. 2:**
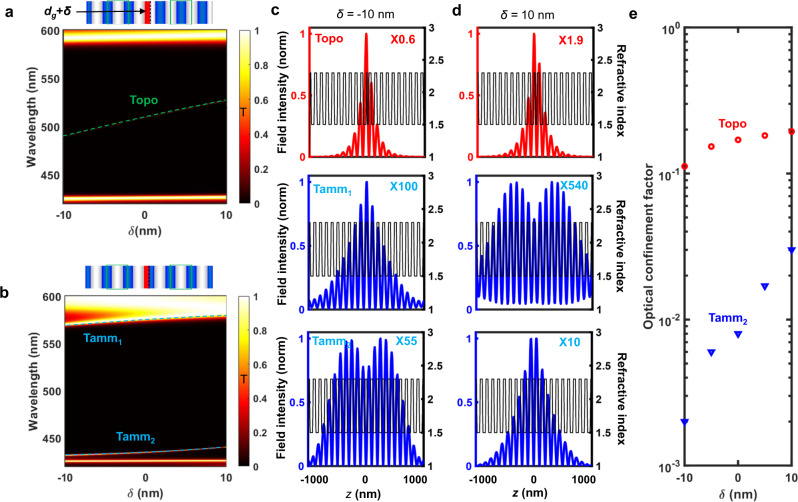
Robustness of the 1D topological microcavity. Calculated transmission spectra of **a** the proposed topological cavity and **b** a trivial cavity upon variation of the first HI layer thickness by $-10\,{nm}\le \delta \le 10\,{nm}$. Electric field distributions of the Tamm and topological states inside the cavity when **c** $\delta=-10{{{{{\rm{nm}}}}}}$ and **d** $\delta=10{{{{{\rm{nm}}}}}}$. **e** Influence of local perturbations on the optical confinement of the topological and Tamm states.

For comparison, we have considered the case of a 1D trivial photonic cavity without topological protection, where PC1 has *d*_*1*_ = 62 nm, *d*_*2*_ = 70 nm and PC2 has *d*_*3*_ = 70 nm, *d*_*4*_ = 62 nm (Supplementary Fig. 1). From Eq. 1, both first optical bands of PC1 and PC2 carry the same topological invariant (Zak phase of $0$). Thus, a topological interface state does not exist within the first bandgap of such 1D cavity. Instead, this structure supports two well-known trivial Tamm states which are also localized near the central interface^24,25^. Based on the calculated transmission spectra, both trivial and topological states show a spectral shift upon variation of the HI layer thickness near the interface by −10 nm ≤ *δ* ≤ 10 nm (Fig. 2a, b). The field distributions in the 1D trivial cavity, however, change significantly while the topological state remains highly confined within the first lattice (Fig. 2c, d). Moreover, compared to the topological state, the trivial Tamm states experience significant changes and reduction in the overall field intensity.

To quantitively evaluate the robustness of the topological states in terms of field confinement near the interface, the optical confinement factor *Γ* of the cavity is defined as^26^:

2 $$\varGamma=\frac{{\int }_{{{{{{\rm{active}}}}}}}{\varepsilon }_{p}{|{{{{{\bf{E}}}}}}(z)|}^{2}{{{{{\rm{d}}}}}}z}{{\int }_{{{{{{\rm{all}}}}}}}\varepsilon {|{{{{{\bf{E}}}}}}(z)|}^{2}{{{{{\rm{d}}}}}}z}$$

Figure 2e clearly shows that the optical confinement factor of the topological cavity is much larger than that of the trivial cavity and it is barely influenced by the variation of the HI layer thickness near the central interface. Conversely, the confinement factor of the trivial cavity varies by more than one order of magnitude when the thickness of the first HI layer changes by$\,-10{nm}\le \delta \le 10\,{nm}$ ($-40\%\le \delta /d\le 40\%$). A similar behaviour is observed when varying the refractive index n_g_ of the hypothetical gain medium (Supplementary Fig. 2).

Overall, the stronger confinement of the topological state near the central interface and its insensitivity to local perturbations compared to trivial Tamm states provide substantial evidence of topological protection in our design. This is ideal for a laser where the first HI layer near the central interface is substituted by a gain medium, provided that sufficient overlap between the optical gain and the topological state is ensured.

**Fig. 3:**
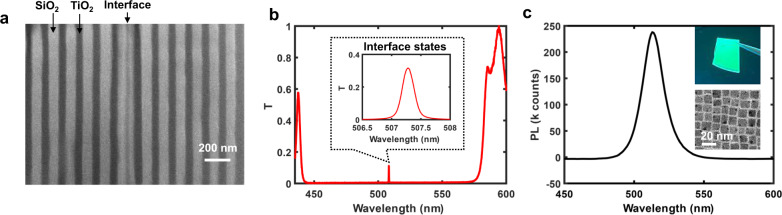
Topological microcavity and gain medium. **a** Cross-sectional scanning electron microscope (SEM) image of the 1D microcavity consisting of 20 alternating layers of TiO_2_ and SiO_2_ and on each side of the interface. **b** Measured transmission spectrum of the 1D topological cavity. The high-Q transmission peak (Q~2000) at 507.3 nm, arising from the interface state, is magnified in the inset. **c** Room temperature photoluminescence spectrum of the CsPbBr_3_ quantum dots film deposited on PC1. The insets show a photograph of the film on PC1 under UV lamp illumination and a transmission electron microscope image of the cuboid CsPbBr_3_ quantum dots, with an average size of 9 nm.

In experiments, we have first verified the existence of the topological interface state by measuring the transmittance of a microcavity made of alternating layers of TiO_2_ (*n*~2.3) and SiO_2_ (*n*~1.5), shown in Fig. 3a. The measured transmission spectrum (Fig. 3b) is in good correspondence to the calculated one, with a high-quality factor interface state (Q~2000) appearing within the optical bandgap at *λ* = 507.3 nm.

To further realize the topological laser, we used solution-processed all-inorganic cesium lead bromide (CsPbBr_3_) quantum dot films (Supplementary Fig. 3) to substitute the HI layer of PC1 near the interface. We synthesized CsPbBr_3_ nanocrystals with nominal size of ~ 9 nm and isotropic spontaneous emission centered around *λ* = 515 nm (Fig. 3c and Supplementary Fig. 6) and cast films with thickness around 45 nm and refractive index *n*~2 on the PC1 side (Supplementary Fig. 4), then mechanically bonded to the complementary PC2 side.

### Lasing in the topological interface state

**Fig. 4:**
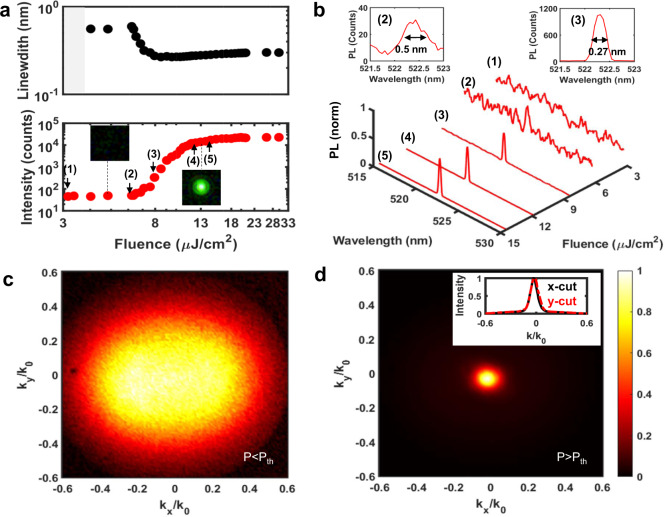
Laser performance of the 1D topological microlaser at room temperature. **a** Light-light curve of the microlaser and linewidth of the emission spectra as a function of the pump fluence at room-temperature. The shadow region indicates the undefined linewidth due to low pump fluence. The insets illustrate representative images of the beam profiles measured at the sample surface, below and above lasing threshold. **b** Room-temperature emission spectra of the topological microlaser as a function of pump fluence, showing laser peaks forming above threshold. The insets show the PL emission spectra obtained at pump fluence of 8.5µJ/cm^2^ (above the lasing threshold) and 6.4 µJ/cm^2^ (below the lasing threshold). Cross-sectional image of the microlaser beam profile measured **c** below and **d** above lasing threshold in the momentum space, where **k**_**x**_*/***k**_**0**_ = *sinθ* and *θ* is the emission angle. The inset in **d** shows the vertical and horizontal directivity of the laser beam.

The performance of the complete laser structure was characterized under λ = 400 nm frequency-doubled fs-laser pump, with 100 fs pulse duration and 1 kHz repetition rate and pulse energy in the range of ~30-300 pJ: the dependence of emission intensity on pump fluence is shown in Fig. 4a. The broad emission spectrum observed at low pump fluences is overtaken by a single, narrow lasing peak (λ = 522.3 nm, FWHM = 0.27 nm) at higher pump fluences (Fig. 4b), indicating transition from the spontaneous to the stimulated emission regime. The S-shaped light-light curve, accompanied by significant narrowing of the linewidth, is consistent with laser emission, with corresponding lasing threshold of 6.8 µJ/cm^2^ (i.e., pulse energy of 73 pJ). The laser emission outcouples as a well-defined Gaussian beam, propagating along the normal to the sample plane. The divergence of the beam dramatically reduces from *θ* = 24° (${{{{{{\bf{k}}}}}}}_{{{{{{\bf{x}}}}}}}/{{{{{{\bf{k}}}}}}}_{{{{{{\bf{0}}}}}}}=0.4$) below threshold (Fig. 4c) to *θ* = 4.6° (${{{{{{\bf{k}}}}}}}_{{{{{{\bf{x}}}}}}}/{{{{{{\bf{k}}}}}}}_{{{{{{\bf{0}}}}}}}=0.08$) above threshold (Fig. 4d), confirming the good directivity and spatial coherence of the topological laser. Spectral narrowing, blue shift, and divergence collapse around the normal direction, experienced by the PL upon crossing the lasing threshold, is evident in the angle resolved maps (Supplementary Fig. 7). We note that the perovskite quantum dot topological laser has comparable lasing performance to a previously reported multimode VCSEL (Supplementary Table 1), which employed a hundred times thicker CsPbBr_3_ quantum dot film (*d*_*g*_ = 4 μm) as the gain medium^18^.

### Tunable topological interface-state cavity lasers

**Fig. 5:**
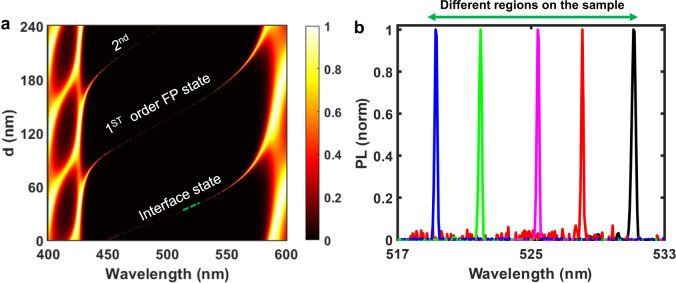
Tunable topological interface-state cavity lasers. **a** Calculated transmission spectra of the 1D topological cavity as function of the thickness of the first HI layer next to the interface (*d*). **b** Tunable single-mode laser emission wavelength obtained by exciting different regions of the sample which correspond to different thicknesses on the perovskite quantum dot film.

When plotting the calculated transmission spectra of the topological cavity as a function of the first HI layer thickness, *d* (Fig. 5a), we find that the interface state survives within the optical bandgap barring a wavelength shift. In combination with the high immunity of field confinement to local perturbations, stable laser wavelength tuning can thus be achieved by selecting different parts of the sample with continuous variation of gain medium thickness. As shown in Fig. 5b, we experimentally confirmed that the topological laser maintains single-mode operation (from *λ* = 532 to 519 nm) excited in different areas of the microlaser, corresponding to a 7 nm variation of gain medium thickness.

Moreover, it has been theoretically suggested that the cavity modes of VCSELs^18,19,27^ are mathematically equivalent to topological surface or edge states^28^. The thickness of the gain medium (including a spacer layer^29^) in such a Fabry–Pérot cavity laser is bound to be an integer multiple *N* of half the emission wavelength ($d=\frac{{\lambda }_{g}}{2}N$*, N* = *1,2,3…*) to fulfill the condition of constructive interference of the resonance in the cavity. Thus, the practical realization of an actual topological interface state in physically thick Fabry–Pérot cavities, remains puzzling. Our multilayer structure allows increasing the thickness of the first HI layer to evolve from the topological interface states into a first-order or higher order Fabry–Pérot state, which is equivalent to adding an extra HI layer with additional n*$\pi$ phase shift near the interface (Supplementary Fig. 8). This extra layer would not change the Zak phase difference between the two PCs. Thus, the topological state still exists in the Fabry–Pérot cavity as a standing wave (Supplementary Table 2), and topological protection is preserved (Supplementary Fig. 9). Although the overall optical confinement in the Fabry–Pérot cavity is higher due to the thicker central HI layer, the confinement per unit gain medium thickness is only about half of the topological cavity, reflecting the high lasing efficiency brought about by the topological interface state.

To compare the lasing action of the 1^st^ order Fabry–Pérot state experimentally, we embedded a 145 nm thick CsPbBr_3_ quantum dot film and pumped the laser cavity under the same conditions used for the topological interface state laser, at room temperature. Both lasers have similar lasing behavior in terms of mode distribution, lasing threshold, and beam profile (Supplementary Table 2). The interface-state laser has a threshold of 6.8 µJ/cm^2^ and saturates slightly earlier than the Fabry–Pérot laser, with a threshold of 9 µJ/cm^2^. Considering that the gain threshold usually increases when decreasing the thickness of the gain medium^30^, the good performance of our microlaser demonstrates the advantage in device miniaturization (reduction of gain medium thickness) brought about by the topological interface cavity design.

## Discussion

The concept of topology, which in recent years has branched from solid-state materials to photonics, has provided a powerful degree of freedom for the manipulation of light and has been successfully used to design robust optical cavities underpinning topological micro- and nano-lasers operating in the infrared. In this work, we have implemented a topological laser based on interface states between two 1D photonic crystals with distinct Zak phases. The 1D topological cavity design, where the thickness of the gain medium can be arbitrarily small, is particularly suitable for the realization of quantum dots lasers, as it allows overcoming issues of poor uniformity, clustering, and luminescence quenching typical of thick quantum dot films.

Specifically, we have shown that the 1D topological cavity has higher field confinement per unit length of gain medium thickness, which allows device miniaturization without increasing the lasing threshold. Using CsPbBr_3_ perovskite quantum dots, which combine excellent optical performances (large optical absorption cross section and exciton binding energy, high photoluminescence quantum yield, very low ASE threshold) and chemical versatility (facile solution processing, controllable film thickness, and morphology, wavelength tuning down to the blue spectral region), we have demonstrated a lithography-free, single-mode topological laser emitting in the green. The emission wavelength could be finely tuned by varying the thickness of the QD film within the dispersion of the interface mode. By further increasing the gain medium thickness, we were able to experimentally trace the evolution of the topological interface state into higher-order Fabry–Pérot modes, showing that VCSELs, where thicker gain media provide additional n*$\pi$ phase shift near the interface, preserve a topological character.

Overall, thanks to the simple and low-cost architecture, the significant reduction in gain medium thickness, and the versatility of colloidal perovskite quantum dots, this work paves the way to the realization of efficient topological lasers operating across the entire visible spectrum.

## Methods

### Synthesis of CsPbBr_3_ quantum dots

Cesium carbonate (Cs_2_CO_3_, 99.9% trace metals basis), lead (II) bromide (PbBr_2_, 98%), oleylamine (OLA technical grade, 70%) and n-octane (anhydrous, ≥99%) were purchased from Sigma-Aldrich. Octadecene (ODE, technical grade 90%) and oleic acid (OA, technical grade 90%) were purchased from Alfa Aesar. For the synthesis of Cs-oleate, 0.326 g of Cs_2_CO_3_, 18 mL of ODE and 1 mL of OA were loaded into a 100 mL three-neck flask. The mixture was heated under vacuum at 100 °C for an hour and subsequently raised to 150 °C under nitrogen flow. Upon complete dissolution of Cs_2_CO_3_, the solution was kept at 150 °C to avoid solidification. For the synthesis of CsPbBr_3_ quantum dots, 0.067 g of PbBr_2_, 5 mL of ODE, 0.5 mL of OA and 1 mL OLA were loaded into a separate 100 mL three-neck flask. The mixture was heated under vacuum at 100 °C for an hour and subsequently raised to 150 °C under nitrogen flow. After the complete dissolution of PbBr_2_, the solution was further heated to 170 °C. 0.6 mL of the as-prepared Cs-oleate was quickly injected and the solution was immediately placed into an ice—water bath and cooled to room temperature. To purify the resulting solution, ethyl acetate was subsequently added into the solution and the solution was centrifuged at 1613g for 5 min. The precipitate was collected and dispersed in n-octane. The solution was again centrifuged at 6000 rpm for 5 min and the supernatant was stored in a nitrogen-filled environment to prevent degradation of the solution.

### Structural and morphological characterization

Transmission electron microscopy images were acquired on a JEM-1400 flash electron microscope operating at 100 kV. Samples were prepared by drop casting the solution onto a 400 mesh copper grids with carbon supporting films. Atomic force microscopy images were acquired on a Cypher ES microscope. Samples were prepared by spin coating the solution onto a glass microscope substrate.

### Device fabrication

The topological DBR mirrors were cleaned sequentially with acetone, 2-propanol, and deionized water under sonication for 5 min each. For the fabrication of the quantum dots films, the CsPbBr_3_ quantum dots solution was spin-coated onto the cleaned DBR mirror at 4000 rpm for 60 s. Subsequently, the other DBR mirror was bonded to the as-coated mirror with epoxy applied at the edges and pressed with a heavy load to reduce the presence of air gaps. The device was left to dry in a nitrogen-filled environment for 24 h.

### Device characterization

To prove the existence of a topological interface state inside the optical bandgap of the PC structure, transmission spectra were measured in an optical microscope with white light illumination. The condenser in the illumination path was adjusted to *NA* of 0.1. Spectra were acquired in the range of 430–600 nm using a spectrometer with a resolution of 0.2 nm (Acton Monochromators SP2300).

To estimate the Q-factor of the passive cavity, high-resolution transmission spectra around the interface state resonance were measured in a home-made setup. The sample was illuminated by a collimated supercontinuum fiber laser (Fianium WL-SC400-8) to minimize the influence of incidence angles. The transmitted signal was detected by an OSA spectrometer (Yokogawa AQ6374) with resolution 0.05 nm.

Photoluminescence of the metasurface was measured by a frequency doubled Ti:Sapphire laser (400 nm, using a BBO crystal) from a regenerative amplifier (repetition rate 1 kHz, pulse width 100 fs, seeded by Mai Tai, Spectra-Physics) The pumping laser was focused by a convex lens (with focus length of 3 cm) onto the top surface of the sample and the spot size on the sample is about 37 µm (Supplementary Fig. 5). Emitted light and corresponding fluorescence microscopy image were collected on the backside of the metasuface by a 5X objective lens aligned with a CCD (Andor iDus 420) coupled spectrometer (Acton IsoPlane SCT 320 with resolution of 0.05 nm) and a camera, respectively. An attenuator and an energy meter were used to tune and measure the pumping density.

Angle-resolved PL spectra and the back focal plane (BFP) imaging were obtained using an inverted optical microscope (Nikon Ti-U), a spectrograph (Acton IsoPlane SCT 320 with a resolution of 0.05 nm), and a charged-coupled detector (CCD, Andor iDus 420). A lens system between the microscope and the spectrograph was used to project the back focal plane of the collection objective (Nikon ×50, *NA* = 0.6) onto the slit of the spectrograph. For back focal plane (BFP) imaging, the slit was fully open, and the grating was set to the zeroth order.

### Calculations and numerical simulations

The transmission spectra were calculated by an analytical transfer matrix method^31^.

The spatial electric field distribution was obtained by FDTD simulations with a commercial software package (Lumerical). Perfect-matched layers were used as boundaries. The 1D topological cavity was excited by a plane wave from one side and the transmission is detected by a monitor on the other side.

The Q-factor was estimated from the transmission spectra as $Q=\,\frac{{\lambda }_{0}}{\triangle \lambda }$, where ${\lambda }_{0}$ is the resonant wavelength and $\triangle \lambda$ is the full-width half-maximum of the resonance.

### Reporting summary

Further information on research design is available in the Nature Portfolio Reporting Summary linked to this article.

## Supplementary information

Supplementary Information

Lasing Reporting Summary

## Data Availability

The authors declare that all data supporting the findings of this study are available within this article and its supplementary information and are openly available in NTU research data repository DR-NTU (Data) at: 10.21979/N9/6JYIIR. Additional data related to this paper may be requested from the authors.
