# Supplementary material for: Perovskite quantum dot one-dimensional topological laser
Source: Nat Commun. 2023 Mar 15;14:1433. doi: 10.1038/s41467-023-36963-6 (PMC10015034; doi:10.1038/s41467-023-36963-6)
Supplement: Supplementary file 1 — Supplementary Information [file 41467_2023_36963_MOESM1_ESM.pdf]

Supporting Information

Perovskite quantum dot one-dimensional topological laser

Jingyi Tian,[1,2 †,*] Qi Ying Tan,[1,3 †] Yutao Wang,[1,3] Yihao Yang,[1,2,#4] Guanghui Yuan,[1,2,#5] Giorgio Adamo,[1,2] Cesare Soci[1,2,*]

[1] *Centre for Disruptive Photonic Technologies, TPI, Nanyang Technological University, 21 Nanyang Link, 637371, Singapore*

[2] *Division of Physics and Applied Physics, School of Physical and Mathematical Sciences, Nanyang Technological University, 637371, Singapore*

[3] *Energy Research Institute @NTU (ERI@N), Interdisciplinary Graduate School, Nanyang Technological University, 50 Nanyang Drive, 637553, Singapore*

Correspondence: jingyi.tian@ntu.edu.sg, csoci@ntu.edu.sg

†These authors contributed equally to this work.

[#4] *Present address:* College of Information Science and Electronic Engineering, Zhejiang University, Hangzhou, China.

[#5] *Present address: Department of Optics and Optical Engineering, University of Science and Technology of China, Hefei, China.*

Table of Contents

Supplementary Note 1 - Band structure of binary one-dimensional photonic crystals 2

Supplementary Note 2 - Robustness of interface state in the 1D topological microcavity 3

Supplementary Figure 1. Trivial 1D photonic cavity 3

Supplementary Figure 2. Robustness of the 1D topological microcavity 3

Supplementary Note 3 - Morphology of $CsPbBr_3$ quantum dots films 4

Supplementary Figure 3. PL spectra of $CsPbBr_3$ QD film and bulk $CsPbBr_3$ film 4

Supplementary Figure 4. Morphology of $CsPbBr_3$ quantum dots films 5

Supplementary Note 4 – Pump beam profile 6

Supplementary Figure 5. Pump beam profile 6

Supplementary Note 5 - Performance of the proposed topological microlaser 7

Supplementary Figure 6. PL from the QD film 7

Supplementary Figure 7. Angular responses of the topological laser 7

Supplementary Table 1. Comparison with perovskite VCSEL [Ref. 19] 8

Supplementary Note 6 - Robustness of Fabry-Pérot states in the VCSEL 9

Supplementary Figure 8. A VCSEL based on Fabry–Pérot resonances 9

Supplementary Figure 9. Robustness of a Fabry–Pérot cavity VCSEL 10

Supplementary Note 7 - Performance of vertical-cavity surface-emitting laser 11

Supplementary Table 1. Comparison between topological interface state and Fabry–Pérot state lasers 12

SUPPLEMENTARY NOTE 1 - BAND STRUCTURE OF BINARY ONE-DIMENSIONAL PHOTONIC CRYSTALS

The band structures of a single binary PC can be calculated as follows,

$$\cos\big(k(d_1 + d_2)\big) = cosk_1d_1cosk_2d_2 - \frac{1}{2}\left(\frac{z_1}{z_2} + \frac{z_1}{z_2}\right)sink_1d_1sink_2d_2, \qquad (1)$$

where $k_i = \omega n_i/c$, $n_i = \sqrt{\mu_i\varepsilon_i}$, $z_i = \sqrt{\mu_i/\varepsilon_i}$, (i = 1, 2), *k* is the Bloch wave vector, *c* denotes the speed of light in vacuum, and ε_i and μ_i are the relative permittivity and permeability in the corresponding media, respectively.

SUPPLEMENTARY NOTE 2 - ROBUSTNESS OF INTERFACE STATE IN THE 1D TOPOLOGICAL MICROCAVITY

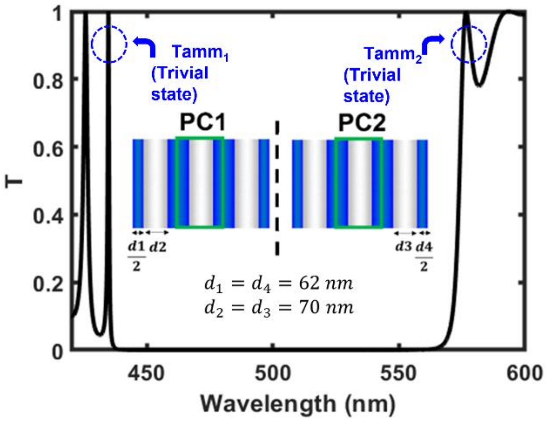

Supplementary Figure 1. Trivial 1D photonic cavity. Calculated transmission spectra of a trivial 1D photonic cavity, where PC1 with d_1=62 nm, d_2=70 nm and PC2 with d_3=70 nm, d_4=62 nm. An interface state does not exist within the first bandgap of such 1D topological cavity.

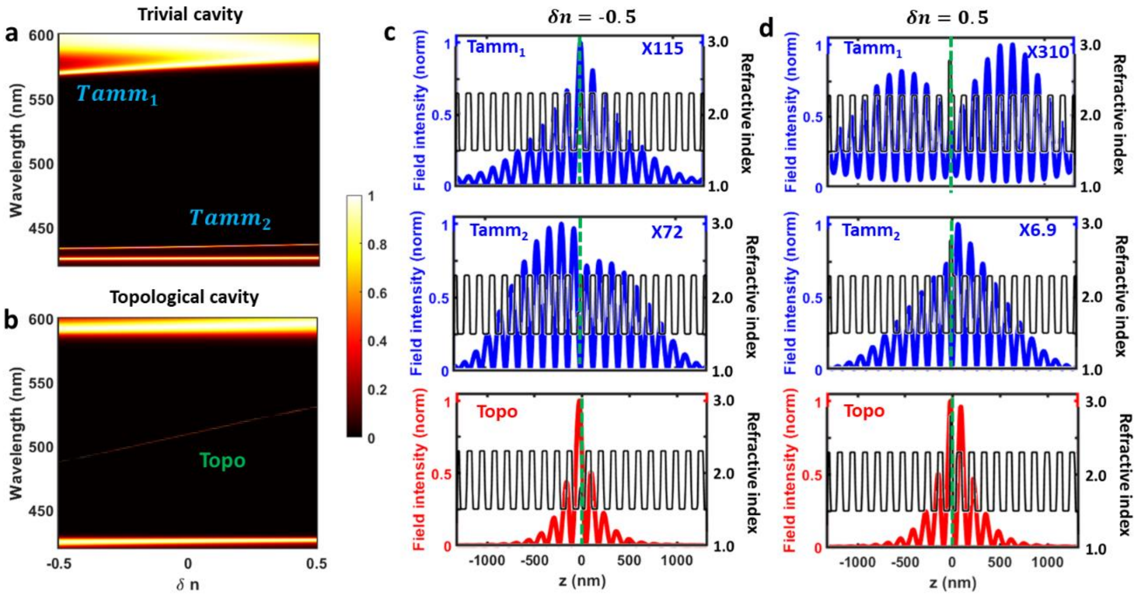

Supplementary Figure 2. Robustness of the 1D topological microcavity. Calculated transmission spectra of (a) the proposed topological cavity and (b) the trivial cavity upon variation of the first HI layer refractive index by $-0.5 \leq \delta n \leq 0.5$. Electric field distributions of the Tamm and topological states inside the cavity when the refractive index of the first HI layer near the interface is changed by (c) $\delta\text{n} = -0.5$ and (d) $\delta\text{n} = 0.5$.

SUPPLEMENTARY NOTE 3 - MORPHOLOGY OF $CsPbBr_3$ QUANTUM DOTS FILMS

Below we compare the PL emission from a film of CsPbBr3 quantum dots (QDs) to that from a bulk $CsPbBr_3$ film. Supplementary Figure 3 shows a clear spectral blue shift of the PL peak for the $CsPbBr_3$ QD film (black curve) with respect to the PL peak for the bulk $CsPbBr_3$ film (shaded orange curve).

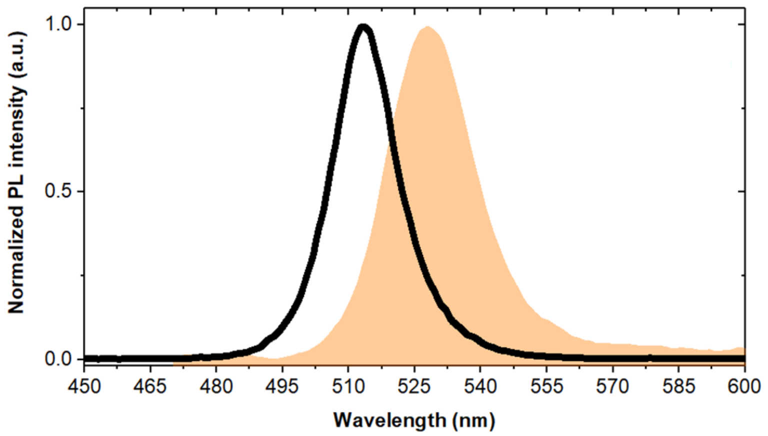

Supplementary Figure 3. PL spectra of the $CsPbBr_3$ QD film (black curve) and the bulk $CsPbBr_3$ film (shaded orange curve).

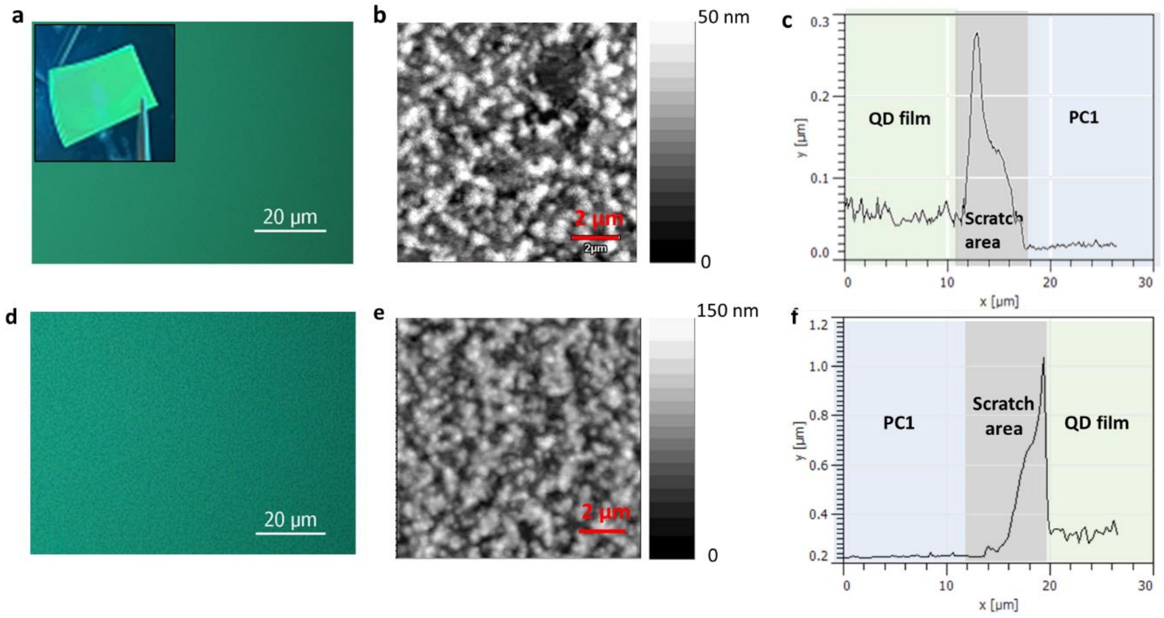

Supplementary Figure 4. Morphology of $CsPbBr_3$ quantum dots films. (a) Optical microscope image of a 45 nm thick $CsPbBr_3$ QD film cast on the PC1, indicating homogeneous surface condition. Inset: $CsPbBr_3$ QD thin film on PC1 under UV lamp illumination. (b) Atomic force microscope (AFM) image of the 45 nm thick $CsPbBr_3$ QD film showing a root mean square roughness of 15 nm. (c) AFM line scan of the 60nm thick $CsPbBr_3$ QD film. (d) Optical microscope image of a 145 nm thick $CsPbBr_3$ QD film cast on the PC1, indicating homogeneous surface condition. (e) AFM image of the 145 nm thick CsPbBr3 QD film showing a root mean square roughness of 18.8 nm. (f) AFM line scan of the 145nm thick $CsPbBr_3$ QD film.

SUPPLEMENTARY NOTE 4 - PUMP BEAM PROFILE

The measured pump beam profile on the sample plane recorded by a CCD is shown in Supplementary Figure 5. The waist of the focal spot of the beam's Gaussian profile ($1/e^2$ level of maximum intensity) is ~37 μm. Based on the measured pump beam profile and power-dependent photoluminescence (Fig. 4 in the manuscript), the lasing threshold is estimated to be around 6.8 μJ/cm^2.

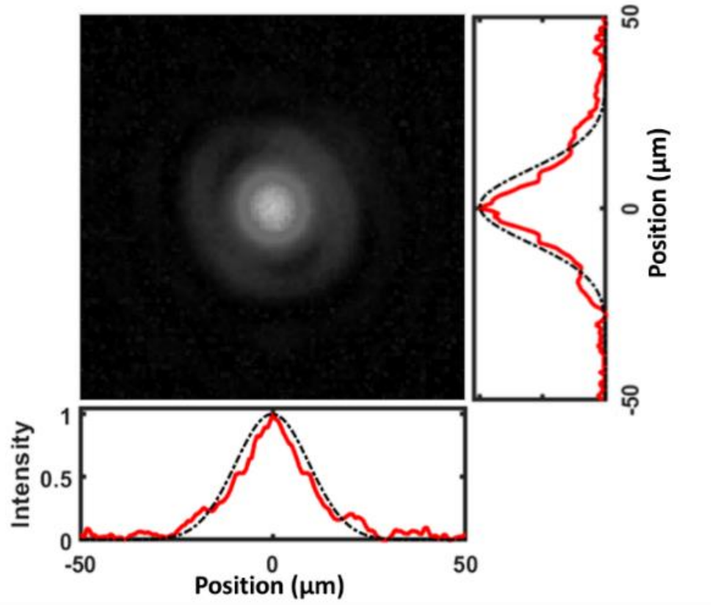

Supplementary Figure 5. Pump beam profile. Cross-sectional image of the pump beam intensity profile measured on the sample surface. The beam is well approximated by a Gaussian profile with waist of 37 μm. The graphs show the beam profile along the vertical and horizontal directions at the sample surface,

SUPPLEMENTARY NOTE 5 - PERFORMANCE OF THE PROPOSED TOPOLOGICAL MICROLASER

The angle-resolved PL map and far-field radiation pattern of the QD film on top of PC1 indicate isotropic emission, as expected (Supplementary Figure 6).

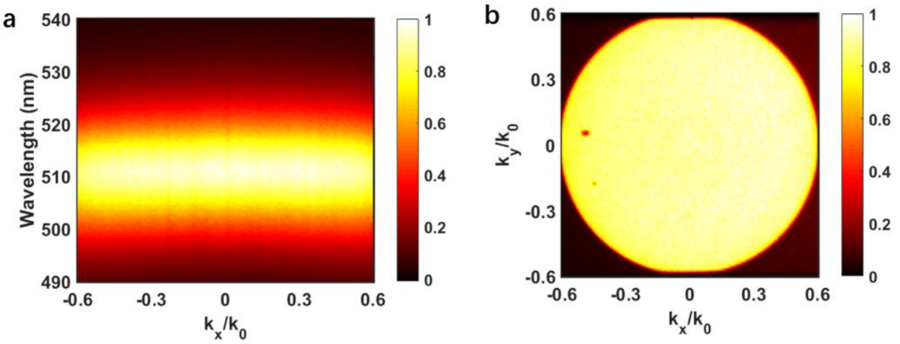

Supplementary Figure 6. PL from the QD film. (a) Angle-resolved PL map and (b) far-field radiation pattern of the QD film on top of PC1 in the momentum space, where $k_x/k_0 = \sin\theta$ and θ is the emission angle.

When the NCs film is integrated in the topological laser cavity the light emission outcouples as a well-defined Gaussian beam. The angular distribution of the emission spectra shows a clear evolution when crossing the laser threshold (Supplementary Figure 7). The bright PL band observable below the lasing threshold (Supplementary Figure 7a), experiences spectral narrowing and blue shift, and an angular collapse around the normal direction (Supplementary Figure 7b) above the lasing threshold.

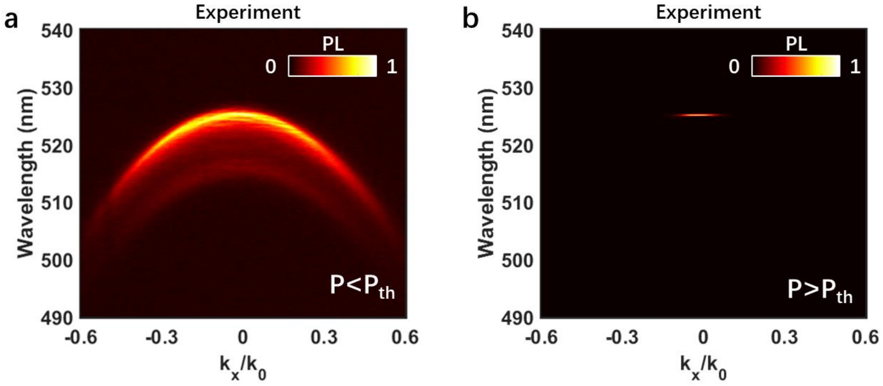

Supplementary Figure 7. Angular responses of the topological laser. Angle-resolved PL map of the NC film integrated in the topological cavity (a) below and (b) above the lasing threshold.

Supplementary Table 1. Comparison of the proposed design with the perovskite VCSEL [Ref. 19]

	Topological cavity perovskite QD laser **[this work]**	Perovskite QD VCSEL **[Adv. Fun. Mater. 27, 1605088 (2017)]**
Design	Topological interface cavity	VCSEL
Gain medium	$CsPbBr_3$ QD film (60 nm thick)	$CsPbBr_3$ QD film (4 µm thick)
Pump condition	100 fs, 1 kHz, 400 nm	100 fs, 1 kHz, 400 nm
Lasing threshold	6.8 µJ/cm^2	11 µJ/cm^2
Single mode	Y	N
Directivity (diverging angle)	4.6 ^{o}	3.6 ^{o}

SUPPLEMENTARY NOTE 6 - ROBUSTNESS OF FABRY-PÉROT STATES IN THE VCSEL

The 1D topological cavity becomes a Fabry–Pérot cavity of the nth order by increasing the thickness of the first HI layer (equivalent to having an extra HI layer with additional $n*\pi$ phase shift), as shown in Supplementary Figure 8. This does not alter the Zak phase difference between the two PCs, thus preserving the topological state that evolves from an interface state to a standing wave, conventionally interpreted as a Fabry–Pérot resonance.

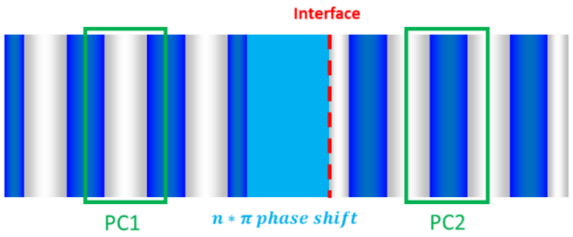

Supplementary Figure 8. A VCSEL based on Fabry–Pérot resonances. By increasing the thickness of the first HI layer, the 1D topological cavity evolves into a Fabry–Pérot cavity, which is equivalent to adding an extra HI layer with additional n*π phase shift (light blue region).

The topological interface state and the 1st order Fabry–Pérot cavity mode have similar intensity and confinement factors and are equally insensitive to local perturbations, as shown in Supplementary Figure 9 where the interface layer thickness is varied by $-10\text{nm} \leq \delta \leq 10\text{nm}$. This shows that Fabry–Pérot modes preserves the robustness typical of topological states. Fig. S8e shows that, although the optical confinement in the Fabry–Pérot cavity is higher due to a thicker HI central layer, the confinement per unit gain medium thickness is lower (about half) of that of the topological cavity, which reflects the high lasing efficiency brought about by the topological interface state.

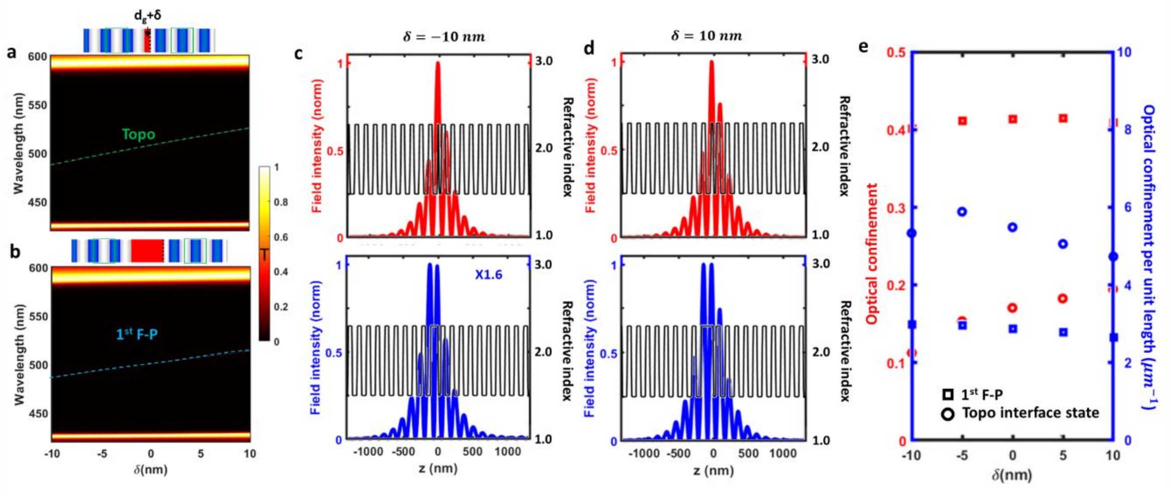

Supplementary Figure 9. Robustness of an Fabry–Pérot cavity VCSEL. Calculated transmission spectra, as function of variation, δ, in the central HI layer thickness for (a) the proposed topological cavity with d= 31 nm and (b) a 1st order Fabry–Pérot VCSEL cavity with d=145 nm. Electric field distributions of the topological (top) and 1st order Fabry–Pérot (bottom) states inside the cavity when the thickness of the first HI layer changes by (c) $\delta = -10$ nm or (d) $\delta = 10$ nm. (e) Influence of local perturbations on the optical confinement of the proposed topological interface state and VCSEL cavities.

SUPPLEMENTARY NOTE 7 - PERFORMANCE OF VERTICAL-CAVITY SURFACE-EMITTING LASER

Lasing at room temperature from a conventional vertical-cavity surface-emitting laser (VCSEL) with thickness of gain medium of 145 nm was characterized under frequency-doubled fs-laser pump (λ = 400 nm), with 100 fs pulse duration and 1 kHz repetition rate.

From the comparison in Supplementary Table 2, it is clear that two structures have similar lasing behaviours. In particular the lasers have:

- Both the topological interface state and the Fabry–Pérot mode are strongly localised inside the layer that will contain the gain medium (row 1).
- Similar linewidth and lasing thresholds: 6.8 μJ/cm^2 for the interface-state laser and of 9 μJ/cm^2 for the Fabry–Pérot laser (row 2 and 3).
- Gaussian beam profiles (row 4).

Supplementary Table 2. Comparison between topological interface state and Fabry–Pérot state lasers.

	Topological interface state	1st F-P state
Mode distribution	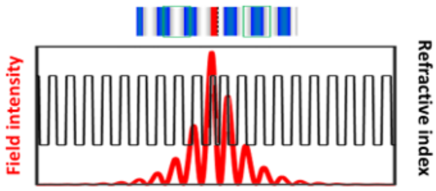	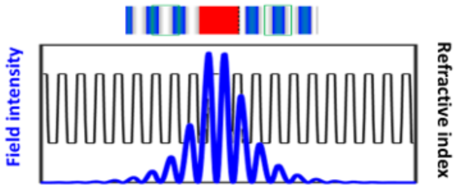
L-L curve (Lasing threshold; Saturation)	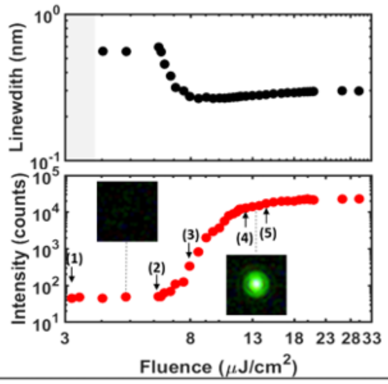	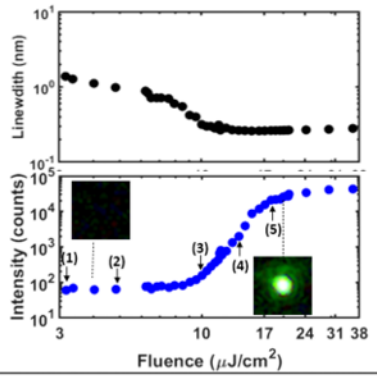
Lasing performance	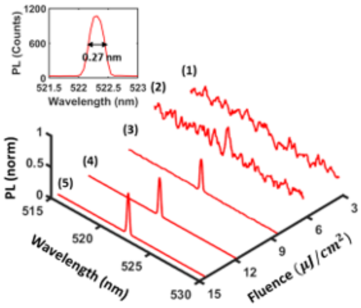	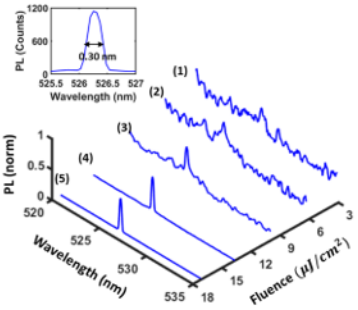
Beam profile at the sample surface	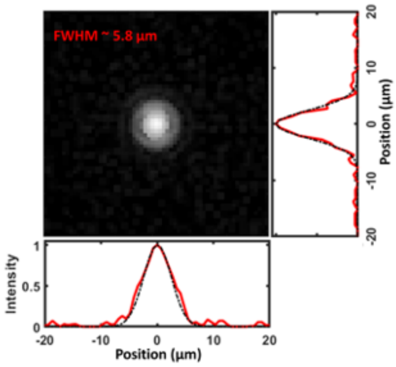	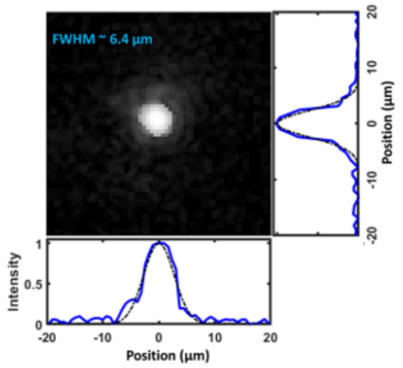
