# Supplementary material for: Perovskite quantum dot one-dimensional topological laser
Source: Nat Commun. 2023 Mar 15;14:1433. doi: 10.1038/s41467-023-36963-6 (PMC10015034; doi:10.1038/s41467-023-36963-6)
Supplement: Supplementary file 2 — Lasing Reporting Summary [file 41467_2023_36963_MOESM2_ESM.pdf]

nature research

Corresponding author(s): Cesare Soci

Lasing Reporting Summary

Nature Research wishes to improve the reproducibility of the work that we publish. This form is intended for publication with all accepted papers reporting claims of lasing and provides structure for consistency and transparency in reporting. Some list items might not apply to an individual manuscript, but all fields must be completed for clarity.

For further information on Nature Research policies, including our data availability policy, see Authors & Referees.

ü Experimental design

Please check: are the following details reported in the manuscript?

1. Threshold

Plots of device output power versus pump power over a wide range of values indicating a clear threshold

- [x] Yes
- [ ] No

Fig. 4a and Fig. 4b and related discussion

2. Linewidth narrowing

Plots of spectral power density for the emission at pump powers below, around, and above the lasing threshold, indicating a clear linewidth narrowing at threshold

- [x] Yes
- [ ] No

Fig. 4b and related discussion

Resolution of the spectrometer used to make spectral measurements

- [x] Yes
- [ ] No

Materials and Methods - 4. Device characterization

3. Coherent emission

Measurements of the coherence and/or polarization of the emission

- [ ] Yes
- [x] No

Unable to measure spatial coherence due to setup limitations

4. Beam spatial profile

Image and/or measurement of the spatial shape and profile of the emission, showing a well-defined beam above threshold

- [x] Yes
- [ ] No

Fig. 4c and Fig. 4d

5. Operating conditions

Description of the laser and pumping conditions
Continuous-wave, pulsed, temperature of operation

- [x] Yes
- [ ] No

Methods - 4. Device characterization

Threshold values provided as density values (e.g. W cm^{-2} or J cm^{-2}) taking into account the area of the device

- [x] Yes
- [ ] No

Fig. 4a and Fig. 4b and related discussion

6. Alternative explanations

Reasoning as to why alternative explanations have been ruled out as responsible for the emission characteristics
e.g. amplified spontaneous, directional scattering; modification of fluorescence spectrum by the cavity

- [x] Yes
- [ ] No

To the best of our knowledge, alternative explanations are inconsistent with the combined observations (1, 2, 4 and 5)

7. Theoretical analysis

Theoretical analysis that ensures that the experimental values measured are realistic and reasonable
e.g. laser threshold, linewidth, cavity gain-loss, efficiency

- [x] Yes
- [ ] No

Simulations in Figs.1-3
Other laser parameters (e.g. laser threshold) in line with previously reported literature results

8. Statistics

Number of devices fabricated and tested

- [x] Yes
- [ ] No

Supplementary Notes 3 and 7

Statistical analysis of the device performance and lifetime (time to failure)

- [ ] Yes
- [x] No

The experiments are intended as proof of principle rather than device performance studies
